# Physical and Mental Health Effects of Bushfire and Smoke in the Australian Capital Territory 2019–20

**DOI:** 10.3389/fpubh.2021.682402

**Published:** 2021-10-14

**Authors:** Rachael M. Rodney, Ashwin Swaminathan, Alison L. Calear, Bruce K. Christensen, Aparna Lal, Jo Lane, Zoe Leviston, Julia Reynolds, Susan Trevenar, Sotiris Vardoulakis, Iain Walker

**Affiliations:** ^1^National Centre for Epidemiology and Population Health, Research School of Population Health, The Australian National University, Canberra, ACT, Australia; ^2^Fenner School of Environment and Society, The Australian National University, Canberra, ACT, Australia; ^3^Departments of General Medicine and Infectious Diseases, The Canberra Hospital, Canberra, ACT, Australia; ^4^Centre for Mental Health Research, Research School of Population Health, The Australian National University, Canberra, ACT, Australia; ^5^Research School of Psychology, The Australian National University, Canberra, ACT, Australia

**Keywords:** bushfire, wildfire, natural disaster, biomass smoke, respiratory health, mental health, Australia

## Abstract

The 2019–20 bushfire season in south-eastern Australia was one of the most severe in recorded history. Bushfire smoke-related air pollution reached hazardous levels in major metropolitan areas, including the Australian Capital Territory (ACT), for prolonged periods of time. Bushfire smoke directly challenges human health through effects on respiratory and cardiac function, but can also indirectly affect health, wellbeing and quality of life. Few studies have examined the specific health effects of bushfire smoke, separate from direct effects of fire, and looked beyond physical health symptoms to consider effects on mental health and lifestyle in Australian communities. This paper describes an assessment of the health impacts of this prolonged exposure to hazardous levels of bushfire smoke in the ACT and surrounding area during the 2019–20 bushfire season. An online survey captured information on demographics, health (physical and mental health, sleep) and medical advice seeking from 2,084 adult participants (40% male, median age 45 years). Almost all participants (97%) experienced at least one physical health symptom that they attributed to smoke, most commonly eye or throat irritation, and cough. Over half of responders self-reported symptoms of anxiety and/or feeling depressed and approximately half reported poorer sleep. Women reported all symptoms more frequently than men. Participants with existing medical conditions or poorer self-rated health, parents and those directly affected by fire (in either the current or previous fire seasons) also experienced poorer physical, mental health and/or sleep symptoms. Approximately 17% of people sought advice from a medical health practitioner, most commonly a general practitioner, to manage their symptoms. This study demonstrated that prolonged exposure to bushfire smoke can have substantial effects on health. Holistic approaches to understanding, preventing and mitigating the effects of smoke, not just on physical health but on mental health, and the intersection of these, is important. Improved public health messaging is needed to address uncertainty about how individuals can protect their and their families health for future events. This should be informed by identifying subgroups of the population, such as those with existing health conditions, parents, or those directly exposed to fire who may be at a greater risk.

## Introduction

During the summer of late 2019 and early 2020, extensive areas of south-eastern Australia experienced one of the most severe bushfire seasons in recorded history. Over 10 million hectares of land were burnt, and over 2,400 homes were destroyed in New South Wales (NSW) alone. Bushfire smoke-related air pollution reached hazardous levels in major metropolitan areas including Canberra, Australia's capital, in the Australian Capital Territory (ACT). Between 15 December and 15 February in the ACT, 27% of days (*n* = 17/63 days) had air quality levels that were considered extremely poor (≥ 300 PM_2.5_ μg/m^3^) and on three quarters of days (*n* = 47/63 days), air quality was classified as poor or worse (≥ 50 PM_2.5_ μg/m^3^) at one or more of Canberra's three air quality monitoring stations (1) [using thresholds defined by NSW Department of Planning, Industry and Environment (2)]. On the worst days, hourly levels reached well over 1000 PM_2.5_ μg/m^3^.

Bushfire is an inevitable and essential part of natural Australian ecosystems; however, the severity and duration of fire seasons are projected to increase globally with climate change (3). A recent study concluded that as a result of anthropogenic climate change, the prevalence of days of high-risk bushfire weather has increased, conservatively, by at least 30% since 1900 (4). As a result of predicted changes to bushfire patterns, the health and community impacts of bushfire are likely to increase unless effective adaptation measures are implemented. The public health impacts of bushfire extend beyond direct exposure to the fire itself, with increasing awareness of the effects of bushfire smoke-related air pollution (5). Smoke can travel long distances and affect large populations, even in urban areas geographically separated from fire (6). Bushfire smoke can have considerable impacts on health; it is estimated 340,000 deaths can be attributed to bushfire smoke globally each year (7). Fine particulate matter, i.e., particles <2.5 micrometers in diameter (PM_2.5_), can adversely affect health via impaired respiratory and cardiac functioning, promotion of inflammation, and alteration of immune function (8, 9). Bushfire-related air pollution has also been associated with increases in mortality (10–12) and the effects of smoke can be more severe in populations with particular vulnerability, such as those with pre-existing medical conditions including cardiovascular disease or respiratory conditions (13, 14).

In bushfire events, it can be difficult to separate the health effects associated with direct displacement, loss, or exposure to fire from those of bushfire smoke. Most studies examining the effects of bushfire smoke on health have used routinely collected health data such as emergency department presentations, hospital admissions and mortality (5), which are very likely to underestimate the effects on the population by capturing only those affected severely enough to require seeking secondary or tertiary-level health care. Prior studies have also focused primarily on physical health concerns, with few studies taking a holistic approach to the assessment of health by including measures of mental health and lifestyle in addition to physical health.

The 2019–20 bushfire season affecting south-eastern Australia elicited widespread public concern due to its duration, and uncertainty about potential immediate and long-lasting health effects of prolonged smoke exposure. In addition, individuals experienced considerable uncertainty about how to protect the health and wellbeing of themselves and their families during this period (15). A preliminary evaluation of the air pollution health burden in eastern Australia estimated that bushfire smoke was responsible for 417 (95% CI, 153–680) excess deaths, over 3,000 excess hospitalizations for cardiovascular (1,124; 95% CI, 211–2,047) and respiratory (2,027; 95% CI, 0–4,252) problems, and 1,305 (95% CI, 705–1,908) presentations to emergency departments with asthma (16). The total smoke-related physical health costs during the 2019–20 bushfire period have been estimated at AU$1.95 billion (17). The 2019–20 bushfires were of particular interest in the Canberra and surrounding community, as this area experienced a severe bushfire in January 2003. During this earlier disaster, almost 160,000 hectares of land were burnt, 448 houses were destroyed (and a much greater number damaged), over 5,000 people were evacuated, four people died and hundreds of people received medical care (18).

This paper presents the findings of a cross-sectional study examining the effects of prolonged exposure to hazardous levels of bushfire smoke-related air pollution on the physical health, mental health, and sleep patterns of residents of the ACT region during the 2019–20 bushfire season and how these vary by demographic and lifestyle factors.

## Materials and Methods

### Study Design

This cross-sectional survey asked participants about their experiences during a “period of interest” defined as 15 December 2019 to 15 February 2020, as this was the period during which the most significant levels of bushfire smoke-related air pollution (hereafter referred to as “smoke”) affected the Canberra region.

### Participant Recruitment

The survey was conducted over a six-week period in March and April 2020. The sampling frame included all adult residents of the ACT and immediately adjacent regions of NSW. Inclusion criteria were: aged 18 years or older, able to understand an online questionnaire in English, and a residential address defined by specific postcode areas constituting the ACT and immediately surrounding postcodes (2600–2612, 2614–2620, 2626, 2900–2906, 2911–2914).

Participants were recruited using three methods: (i) Letter–An invitation to complete the online survey was posted to a random selection of 10 000 addresses, selected from the ACTmapi database (“ACT Addressing” from ACTmapi ©Australian Capital Territory). Any addresses identified as non-residential (e.g., businesses, schools) were removed and replaced prior to the mail out; (ii) Panel–A representative population sample of participants recruited by an external company; (iii) General–A convenience sample of the population was recruited via social media, radio advertisements and word of mouth, including interest from local media.

### Data Collection

Potential participants were invited to complete the online survey via REDCap electronic data capture tools hosted at Australian National University (19). The survey was available in English and in an online format only. Participants provided consent by submitting a completed survey. Two participants that received a letter mailout invitation and who did not have internet access completed the survey by phone with a study team member recording their responses.

The survey collected: postcode of residence, age (years), highest level of education achieved (no school qualification/ school or intermediate/ HSC or leaving certificate/ trade or apprenticeship/ certificate or diploma/ university or higher), tobacco smoking (never/past/current), rental status (renter/owner-occupier/other), pregnancy status (self/partner/none/not applicable), parental (yes/no and age of youngest child) or carer status (yes/no); previous medical diagnoses [asthma/chronic obstructive pulmonary disease (COPD–including emphysema and chronic bronchitis)]/allergies or hay fever/other respiratory disease (e.g., pleurisy, bronchiectasis, pulmonary fibrosis)/multiple sclerosis/arthritis/diabetes/other), engagement with a professional for a mental health concern during the last 12 months (yes/no) and self-rated health (poor/fair/good/very good/excellent); prior exposure to bushfire (no previous exposure to bushfire/been in an area with fire nearby/evacuated due to bushfire/experienced loss of or damage to property/had direct contact with bushfire (e.g.,firefighter or protecting property)/other) including whether this was during the 2003 Canberra bushfire (yes/no); direct exposure to bushfire in the current season (not affected/voluntarily relocated/forced to evacuate/damage to or loss of property/family or close friend affected/had to cancel or alter travel or holiday plans/ firefighter or first responder/other); effects of smoke on physical health (eye irritation or watery eyes/throat irritation or dry throat/cough/wheeze or whistling chest/sneezing/chest tightness or pain/breathlessness/headache/diarrhea or gastroenterological symptoms/other condition not listed), sleep (disrupted or poor sleep/fatigue or feeling tired) and mental health (anxiety/feeling depressed) symptoms and whether these were attributed to smoke (yes/no/unsure); health advice sought as a result of symptoms (yes/no, hospital inpatient/emergency department/general practitioner/specialist/24 h health advice hotline/pharmacist/mental health professional e.g., psychologist/other health professional).

### Data Analysis

#### Data Management

Summary statistics were used to describe the study's participants (Table 1) using demographic, health and lifestyle variables as described in Table 2. To explore physical health outcomes, data were divided into quartiles based on the cumulative number of physical health symptoms reported. A mental health outcome variable was created by including those who self-reported anxiety and/or feeling depressed as a result of smoke. Similarly, a sleep outcome variable was created by combining those who reported disrupted or poor sleep and/or fatigue or feeling tired. Although scaled, validated measures, especially of mental and physical health status, are generally preferable, this paper reports results of a rapid research response developed during the bushfire crisis, with the intention of reporting responses during or soon after the emergency in a way that would be helpful for future researchers and public health officials. Accordingly, our outcomes measures are brief assessments. They were developed from the research team's expertise in different contexts, and are based on our previous research (13, 15, 20).

**Table 1:** Description of sample characteristics.

**Factor**	***n* (%)**
**Gender**	
Male	831 (40.2)
Female	1,231 (59.6)
Other	4 (0.2)
**Age (years)**	45 (SD 16.8) min 18, max 85
18–24	128 (6.1)
25–34	396 (19.0)
35–44	328 (15.7)
45–54	310 (14.9)
55–64	427 (20.5)
65-74	381 (18.3)
75+	113 (5.4)
**Education**	
No school qualification, school or intermediate, HSC or leaving	266 (12.9)
Trade, apprenticeship, certificate or diploma	346 (16.8)
University	1,451 (70.3)
Parent (Yes)	548 (26.5)
Age of youngest child	
0–18 month	113 (20.8)
>18 month−4 years	123 (22.7)
5–11 years	176 (32.4)
12–17 years	131 (24.1)
**Carer** (Yes)	317 (15.4)
**Pregnant** (Self)	43 (2.1)
**Self-rated health**	
**Poor-Fair**	205 (10.6)
**Good-Excellent**	1,730 (89.4)
**Previous diagnosis of a physical health condition** (Yes)	1,241 (62.5)
**Previous diagnosis of a mental health condition** (Yes)	441 (29.4)
**Renter** (Yes)	295 (22.2)
**Smoker**	
Never	1,490 (72.3)
Past	512 (24.9)
Current	58 (2.8)
**Direct fire exposure—current season**	
None	859 (41.7)
Mild	1,032 (50.1)
Severe	168 (8.2)
**Cumulative direct fire exposure in current season** (number of experiences)	
0	880 (42.2)
1	787 (37.8)
2	315 (15.1)
3	88 (4.2)
4	13 (0.6)
5	1 (0.1)
**Previous Fire Exposure**	
None	686 (33.0)
Mild	1,173 (56.5)
Severe	217 (10.5)
**Exposure to 2003 Canberra bushfires** (Yes)	905 (43.4)
Cumulative previous fire exposure (number of experiences)	
0	689 (33.1)
1	1,271 (61.0)
2	98 (4.7)
3	24 (1.2)
4	2 (0.1)

**Table 2:** Details of variables used to describe the study population in regression models.

**Variable**	**Description**
Gender	Due to the small number of “other” responses, gender was included in regression models as binary variable (male vs. female)
Age	Explored as either continuous (years) or categorical (18–24, then 10 year bins (e.g., 25–34) up to 75+ years) variables
Parent	No vs. yes (parent of one or more children under 18 years)
Parent age	Age of youngest child grouped into 0–18 months, under 5 years, 5–11 years, 12–17 years
Carer	No vs. yes (carer of one or more non-child dependents)
Pregnant	No vs. yes (self-pregnant)
Self-rated health	Grouped into two groups; fair and poor vs. excellent, very good and good
Previous physical health diagnosis	No vs. yes (one or more previous diagnoses)
Previous mental health diagnosis	No vs. yes (engagement with a professional for a mental health concern during the last 12 months)
Renter	Two categories–owner-occupier vs. renter
Smokers	Three categories–never, past, current
Education	The level of education completed was grouped into: no school qualification, school or intermediate, HSC or leaving; vs. Trade or apprenticeship, certificate or diploma; vs. university or higher
Direct fire exposure	Direct exposure to fire in the current season was measured as: 1. Any exposure—yes/no if any direct exposures to fire were indicated; 2. Scale—a scale was created consisting of three levels of exposure—none (none or indirect), mild (classified as responses limited to being in an area with fire nearby, evacuation due to bushfire, area of significance lost other than home, family member was affected, home was affected while away), and severe (if experience included loss of or damage to property or direct contact with fire e.g., firefighter or protecting property); 3. Cumulative—the number of ways in which the participant has previously been exposed for fire were added
Previous fire exposure	Previous exposure to fire was measured as: 1. Any exposure—yes/no if any previous exposures were indicated; 2. Scale—a scale was created with three levels of exposure—None (not affected, effects were limited to health and/or smoke effects), mild (responses limited to voluntary evacuation, family or close friend affected, cancellation or alteration of holiday plans/events, business or work affected), and severe (if experience included forced evacuation, damage to or loss of property, firefighter, first responder, volunteer, protected property, alert to evacuate, worry about property or risk);Cumulative—The number of ways in which the participant has previously been exposed for fire were added
2003 fires	For those that indicated previous exposure to fire, no (exposure was a result of another fire event) vs. yes (experience was as a result of 2003 fire in Canberra)

#### Statistical Analysis

All analyses were conducted in Stata (v15.1, College Station, TX StataCorp LLC). Summary statistics were used to describe the study sample as detailed in Table 1. Number (%) were used to describe categorical variables and mean (SD) to describe continuous data. Descriptive statistics were calculated to examine the prevalence of self-reported physical health, sleep and mental health symptoms, overall and for men and women which were compared using Chi^2^ analyses. Ordinal logistic regression was used to identify factors (as per Table 2) that were associated with higher physical health symptoms. Bivariate analyses including age, gender and age^*^gender interaction were then conducted for factors for which p ≤ 0.1. Logistic regression was used to determine factors that were associated with either disrupted sleep or mental health symptoms. Similar bivariate analyses were conducted to develop sleep and mental health models. Summary statistics [n (%)] were used to describe sources of health advice sought, overall and separately for males and females, which were compared using Chi^2^ analyses.

## Results

### Population

A total of 2,095 completed responses to the survey were received; 644 (30.7%), 639 (30.5%), and 812 (38.8%) people recruited via the panel, letter and general recruitment methods respectively. This was a response rate of approximately 6.4% to the letter invitations, although many were returned to sender so it was unclear how many were undelivered. Eleven responses were excluded as the participant resided outside of the target area or did not provide a valid postcode. This resulted in a total sample of 2,084 responses that were included in subsequent analyses.

Participant characteristics are summarized in Table 1. Of the study participants, 40.2% were male, 59.6% were female, and 0.2% identified as being of another gender. The mean age of respondents was 45 years (median 45, range 18–85 years), approximately 10 years older than the median age of ACT residents. Approximately 13% of the sample had completed a high school certificate or less, 17% a trade, apprenticeship, certificate or diploma, and 70% had completed a University degree. This suggests that the sample included a greater proportion of women, was on average older, and more highly educated than the ACT population compared with data from the Australian Bureau of Statistics (21).

Approximately a quarter of respondents were parents, 15.4% were carers for one or more non-child dependents and 22% of participants rented their place of residence. Forty-three respondents (2.1%) were pregnant during the period of interest, 11% rated their own health as poor-fair (vs 89% good-excellent), 3% were current smokers, 25% were ex-smokers and 72% had never smoked. Over half of the sample (58%) had been directly affected by fire (not just smoke) during the 2019–20 bushfire season, with 8% of the total sample reporting being affected severely. Previous direct exposure to bushfire had been reported by 67% of the sample, 10.5% severely. For most participants (43% of the total sample), this exposure had been during the 2003 Canberra bushfire event.

### Physical Health

Most participants (97.1%) reported experiencing at least one specified physical health symptom during the period of interest that they attributed to the smoke (Table 3). The most common symptoms were eye irritation or watery eyes (73.1%), throat irritation or dry throat (70.4%) and cough (50.6%). A considerable proportion of people also reported experiencing headaches (38.2%), breathlessness (21.9%), sneezing (21%) or wheeze/whistling chest (19.9%). A small number of people (3.2%) also reported experiencing other (non-itemized) physical or mental health impacts of the smoke including asthma, bronchitis, itchy skin/rash, runny nose, sinusitis and mental trauma. Women reported all physical symptoms more frequently than men (*p* <0.001 for all other than sneeze *p* <0.05) and had almost three times the odds of men of experiencing a greater number of physical health conditions as a result of exposure to smoke (2.92 ± 0.344, *p* <0.001) (Table 4). After accounting for age and gender, those participants with poorer self-rated health (2.13 ± 0.344, *p* <0.001), a previous diagnosis of either a physical (2.18 ± 0.266, *p* <0.001) or mental health condition (1.64 ± 0.206, *p* <0.001), or were a past (but not current) smoker (1.83 ± 0.226; *p* <0.001), had greater odds of experiencing more physical symptoms. Any direct exposure with fire this season increased odds of experiencing a greater number of health symptoms (1.64 ± 0.186, *p* <0.001). The effect was strongest in those who had experienced more severe effects of bushfire compared to those who reported mild exposure to fire (2.47 ± 0.465 vs. 1.52 ± 0.178; *p* < 0.001). Previous fire exposure increased the odds of experiencing more physical health symptoms, whether this was defined as any previous exposure (1.55 ± 0.182, *p* <0.001), mild or severe exposure (1.51 ± 0.181; *p* = 0.001, 1.82 ± 0.345; *p* = 0.001, for mild and severe respectively) or as a cumulative measure (1.40 ± 0.121; *p* < 0.001). Having experienced the 2003 Canberra bushfires did not statistically increase the odds of experiencing a greater number of negative physical health symptoms (1.56 ± 0.447; *p* = 0.124). Parental, carer, pregnancy or education status were not associated with an increase in physical health symptoms.

**Table 3:** Proportion of surveyed ACT region residents that experienced health-related symptoms attributed to bushfire smoke during the 2019–20 bushfire season.

	**Number experiencing the symptom n (%)**		
**Symptom**	**All**	**Male**	**Female**
	***n* = 2,084**	***n* = 832**	***n* = 1,231**
**Physical symptoms**			
Eye irritation or watery eyes	1,525 (73.1)	544 (65.3)	967 (78.6)
Throat irritation or dry throat	1,469 (70.4)	506 (60.8)	950 (77.2)
Cough	1,056 (50.6)	346 (41.5)	702 (57.0)
Headache	797 (38.2)	184 (22.1)	607 (49.3)
Breathlessness	457 (21.9)	126 (15.0)	326 (26.5)
Sneezing	437 (21.0)	153 (18.4)	278 (22.6)
Wheeze or whistling chest	416 (19.9)	103 (12.3)	307 (24.9)
Chest tightness or pain	314 (15.1)	71 (8.5)	242 (19.7)
Diarrhea or gastroenterological symptoms	51 (2.4)	6 (0.7)	44 (3.6)
Other condition not listed	67 (3.2)	19 (2.3)	47 (3.8)
**Mental health symptoms**			
Anxiety	945 (45.3)	265 (31.9)	670 (54.4)
Depression	447 (21.4)	129 (15.5)	314 (25.5)
**Sleep-related symptoms**			
Disrupted or poor sleep	776 (37.2)	234 (28.1)	535 (43.5)
Fatigue or feeling tired	677 (32.5)	187 (22.5)	482 (39.2)

*Difference between gender P < 0.001 for all other than sneeze P = 0.022, and other P = 0.05*.

**Table 4:** Ordinal logistic regression examining factors associated with odds of greater number of self-reported physical health symptoms attributed to bushfire smoke exposure.

	**Univariate**			**Bivariate^a^**		
	**OR (SE)**	**95%CI**	***P*-value**	**OR (SE)**	**95%CI**	***P*-value**
**Gender**	2.92 (0.344)	2.31–3.68	<0.001			
**Age (years)**	0.99 (0.003)	0.98–1.00	0.001			
18–24	1.00					
25–34	0.23 (0.245)	0.03–1.81	0.165			
35–44	0.26 (0.275)	0.03–2.06	0.203			
45–54	0.35 (0.379)	0.04–2.89	0.332			
55–64	0.41 (0.439)	0.05–3.33	0.405			
65–74	0.32 (0.336)	0.04–2.53	0.278			
75+	0.10 (0.106)	0.01–0.82	0.032			
**Parent**	1.14 (0.130)	0.91–1.42	0.257			
**Age of youngest child**						
0–18m	1.00					
>18 month−4 years	1.42 (0.433)	0.78–2.58	0.255			
5–11 years	1.43 (0.408)	0.82–2.50	0.209			
12–17 years	1.41 (0.424)	0.78–2.54	0.260			
**Carer**	1.17 (0.160)	0.89–1.53	0.266			
**Pregnant**	1.20 (0.412)	0.61–2.35	0.598			
**Self-rated health**	2.05 (0.317)	1.51–2.77	<0.001	2.13 (0.344)	1.56–2.93	<0.001
**Previous physical health diagnosis**	2.20 (0.257)	1.75–2.76	<0.001	2.18 (0.266)	1.72–2.77	<0.001
**Previous mental health diagnosis**	1.73 (0.209)	1.36–2.19	<0.001	1.64 (0.206)	1.28–2.09	<0.001
**Renter**	1.32 (0.187)	1.00–1.75	0.046	1.24 (0.194)	0.92–1.69	0.163
**Smoker**						
Never	1.00					
Past	1.54 (0.170)	1.23–1.93	<0.001	1.83 (0.226)	1.44–2.33	<0.001
Current	1.34 (0.402)	0.74–2.41	0.331	1.50 (0.463)	0.82–2.74	0.193
**Education**						
No school qualification, school or intermediate, HSC or leaving	1.00					
Trade, apprentership, certificate or diploma	1.20 (0.229)	0.84–1.75	0.314			
University	1.05 (0.165)	0.77–1.43	0.757			
**Direct fire^b^**						
Any exposure	1.85 (0.202)	1.49–2.29	<0.001	1.64 (0.186)	1.31–2.04	<0.001
2. Scale–None	1.00					
Mild	1.72 (0.195)	1.38–2.15	<0.001	1.52 (0.178)	1.21–1.91	<0.001
Severe	2.73 (0.497)	1.91–3.90	<0.001	2.47 (0.465)	1.71–3.58	<0.001
3. Cumulative	1.44 (0.080)	1.29–1.60	<0.001	1.35 (0.079)	1.21–1.51	<0.001
**Previous Fire^c^**						
1. Any exposure	1.36 (0.153)	1.09–1.69	0.006	1.55 (0.182)	1.23–1.95	<0.001
2. Scale – none	1.00					
Mild	1.35 (0.156)	1.08–1.70	0.009	1.51 (0.181)	1.19–1.91	0.001
Severe	1.40 (0.252)	0.98–1.99	0.063	1.82 (0.345)	1.26–2.64	0.001
3. Cumulative	1.31 (0.108)	1.11–1.54	0.001	1.40 (0.121)	1.19–1.66	<0.001
Canberra bushfires 2003	1.56 (0.447)	0.89–2.73	0.124			

a Bivariate models included factors along with gender, age and gender^*^age interaction;

b *Direct exposure to fire in the current season was measured as: 1. Any exposure – yes/no if any direct exposures to fire were indicated; 2. Scale—a scale was created consisting of three levels of exposure—none (none or indirect), mild (classified as responses limited to being in an area with fire nearby, evacuation due to bushfire, area of significance lost other than home, family member was affected, home was affected while away), and severe (if experience included loss of or damage to property or direct contact with fire e.g., firefighter or protecting property); 3. Cumulative—the number of ways in which the participant has previously been exposed for fire were added;*

c *Previous exposure to fire was measured as: 1. Any exposure–yes/no if any previous exposures were indicated; 2. Scale–a scale was created with three levels of exposure—None (not affected, effects were limited to health and/or smoke effects), mild (responses limited to voluntary evacuation, family or close friend affected, cancellation or alteration of holiday plans/events, business or work affected), and severe (if experience included forced evacuation, damage to or loss of property, firefighter, first responder, volunteer, protected property, alert to evacuate, worry about property or risk); 3. Cumulative–The number of ways in which the participant has previously been exposed for fire were added*.

### Mental Health

Over 55% of responders self-reported symptoms of anxiety (45.3%) and/or feeling depressed (21.4%) as a result of the smoke (Table 3). Women were more likely than men to report negative mental health outcomes (1.99 ± 0.192, *p* <0.001) which reflected increased symptoms of both anxiety (54.5 vs. 31.8%) and feelings of depression (25.5 vs. 15.5%). Poor mental health outcomes were associated with younger age groups (25–54 years groups). Bivariate models (Table 5) identified parents (1.27 ± 0.142; *p* = 0.029), individuals with an existing physical (1.35 ± 0.138; *p* = 0.004) or mental health diagnosis (1.30 ± 0.164; *p* = 0.038), and those who had obtained a higher level of education (high school or lower vs. trade/diploma: 1.46 ± 0.257, *p* = 0.032 vs university: 1.42 ± 0.208; *p* = 0.015) had greater odds of experiencing negative mental health outcomes (Table 5). Direct exposure to fire in the current season was associated with increased reported symptoms of anxiety and feeling depressed, according to all measures used (p ≤ 0.001). Of note, severe bushfire exposure had a stronger effect on mental health than mild exposure (1.87 ± 0.344 and 1.46 ± 0.148 for severe and mild, respectively). Exposure to fire in a previous season, including during the 2003 Canberra fires, was not associated with mental health outcomes.

**Table 5:** Logistic regression examining factors associated with self-reported negative mental health outcomes attributed to bushfire smoke exposure.

	**Univariate**			**Bivariate^a^**		
	**OR (SE)**	**95%CI**	***P*-value**	**OR (SE)**	**95%CI**	***P*-value**
**Gender**	1.99 (0.192)	1.65–2.40	<0.001			
**Age (years)**	0.99 (0.003)	0.98–0.99	<0.001			
18–24	1.00					
25–34	1.71 (0.365)	1.12–2.59	0.012			
35–44	2.07 (0.455)	1.34–2.18	0.001			
45–54	1.59 (0.350)	1.03–2.45	0.034			
55–64	1.13 (0.239)	0.75–1.72	0.549			
65–74	0.96 (0.205)	0.63–1.46	0.837			
75+	0.70 (0.201)	0.40–1.23	0.212			
**Parent**	1.43 (0.150)	1.16–1.75	0.001	1.27 (0.142)	1.02–1.59	0.029
**Age of youngest child**						
0–18m	1.00			1.00		
>18 month−4 years	1.25 (0.354)	0.72–2.18	0.423	1.26 (0.364)	0.71–2.22	0.426
5–11 years	0.95 (0.242)	0.58–1.57	0.848	0.96 (0.257)	0.57–1.62	0.885
12–17 years	0.60 (0.162)	0.35–1.02	0.058	0.60 (0.173)	0.34–1.06	0.078
**Carer**	0.89 (0.114)	0.69–1.15	0.367			
**Pregnant**	1.51 (0.526)	0.77–2.99	0.234			
**Self-rated health**	1.17 (0.186)	0.86–1.60	0.323			
**Previous physical health diagnosis**	1.32 (0.130)	1.09–1.60	0.004	1.35 (0.138)	1.10–1.64	0.004
**Previous mental health diagnosis**	1.45 (0.178)	1.14–1.85	0.002	1.30 (0.164)	1.01–1.66	0.038
**Renter**	1.12 (0.157)	0.85–1.48	0.409			
**Smoker**						
Never	1.00					
Past	1.06 (0.115)	0.86–1.31	0.592			
Current	0.86 (0.240)	0.49–1.48	0.579			
**Education**						
No school qualification, school or intermediate, HSC or leaving	1.00					
Trade, apprentership, certificate or diploma	1.40 (0.243)	1.00–1.97	0.050	1.46 (0.257)	1.03–2.06	0.032
University	1.37 (0.196)	1.04–1.82	0.026	1.42 (0.208)	1.07–1.90	0.015
**Direct fire^b^**						
1. Any exposure	1.65 (0.157)	1.37–1.99	<0.001	1.51 (0.149)	1.25–1.83	<0.001
2. Scale–None	1.00					
Mild	1.60 (0.157)	1.32–1.94	<0.001	1.46 (0.148)	1.20–1.78	<0.001
Severe	2.02 (0.365)	1.42–2.88	<0.001	1.87 (0.344)	1.31–2.69	0.001
3. Cumulative	1.41 (0.077)	1.26–1.57	<0.001	1.34 (0.076)	1.20–1.50	<0.001
**Previous Fire^c^**						
1. Any exposure	0.96 (0.095)	0.79–1.17	0.683			
2. Scale -None	1.00					
Mild	0.92 (0.094)	0.75–1.12	0.412			
Severe	1.23 (0.205)	0.88–1.70	0.223			
3. Cumulative	1.08 (0.082)	0.93–1.25	0.346			
Canberra bushfires 2003	1.05 (0.097)	0.87–1.26	0.621			

a Bivariate models included factors along with gender, age and gender*age interaction;

b *Direct exposure to fire in the current season was measured as: 1. Any exposure—yes/no if any direct exposures to fire were indicated; 2. Scale—a scale was created consisting of three levels of exposure—none (none or indirect), mild (classified as responses limited to being in an area with fire nearby, evacuation due to bushfire, area of significance lost other than home, family member was affected, home was affected while away), and severe (if experience included loss of or damage to property or direct contact with fire e.g., firefighter or protecting property); 3. Cumulative—the number of ways in which the participant has previously been exposed for fire were added;*

c *Previous exposure to fire was measured as: 1. Any exposure–yes/no if any previous exposures were indicated; 2. Scale–a scale was created with three levels of exposure—None (not affected, effects were limited to health and/or smoke effects), mild (responses limited to voluntary evacuation, family or close friend affected, cancellation or alteration of holiday plans/events, business or work affected), and severe (if experience included forced evacuation, damage to or loss of property, firefighter, first responder, volunteer, protected property, alert to evacuate, worry about property or risk); Cumulative–The number of ways in which the participant has previously been exposed for fire were added*.

### Sleep

Half of survey respondents reported poorer sleep as a result of exposure to smoke (Table 3). This was defined as either disrupted or poor sleep (37.2%) and/or fatigue or feeling tired (32.5%). Females were more likely to experience poorer sleep outcomes than men (1.75 ± 0.168, *p* <0.001). Odds of poor sleep outcomes were increased in younger age groups. When age and gender were accounted for, those with poorer self-rated health (2.01± 0.339; *p* <0.001), or a previous diagnosis of a physical health condition (1.34± 0.136; *p* <0.01) all had greater odds of experiencing smoke-attributed impairments in sleep (Table 6). Direct exposure to fire in the current fire season increased the odds of poor sleep outcomes, irrespective of the measure used (*p* < 0.001). This effect was more pronounced in those who had experienced more severe exposure (1.81 ± 0.326 vs. 1.34 ± 0.135 for mild vs. severe exposure, respectively). The odds of poor sleep outcomes were increased in the group who had experienced more severe exposures to fire in a previous season (1.82 ± 0.312; *p* < 0.001), and tended to be increased to a lesser extent in the group who had more mild experiences (1.29 ± 0.135; p = 0.014).

**Table 6:** Logistic regression examining factors associated with negative self-reported sleep outcomes attributed to bushfire smoke exposure.

	**Univariate**			**Bivariate^a^**		
	**OR (SE)**	**95%CI**	***P*-value**	**OR (SE)**	**95%CI**	***P*-value**
**Gender**	1.75 (0.168)	1.45–2.11	<0.001			
**Age (years)**	0.99 (0.003)	0.98–0.99	<0.001			
18–24	1.00					
25–34	1.69 (0.361)	1.11–2.57	0.014			
35–44	1.47 (0.321)	0.96–2.26	0.075			
45–54	1.51 (0.333)	0.98–2.33	0.059			
55–64	1.15 (0.243)	0.76–1.74	0.516			
65–74	0.89 (0.191)	0.58–1.35	0.579			
75+	0.81 (0.233)	0.46–1.43	0.470			
**Parent**	1.29 (0.133)	1.05–1.58	0.015	1.18 (0.129)	0.96–1.47	0.121
**Age of youngest child**						
0–18 months	1.00					
>18 month-4 years	1.67 (0.451)	0.99–2.84	0.056	1.63 (0.448)	0.95–2.79	0.077
5–11 years	1.68 (0.416)	1.03–2.73	0.036	1.66 (0.430)	0.996–2.76	0.052
12–17 years	1.25 (0.330)	0.75–2.10	0.394	1.29 (0.364)	0.74–2.25	0.363
**Carer**	0.96 (0.123)	0.74–1.23	0.733			
**Pregnant**	1.42 (0.481)	0.73–2.76	0.298			
**Self-rated health**	1.97 (0.324)	1.43–2.72	<0.001	2.01 (0.339)	1.45–2.80	<0.001
**Previous physical health diagnosis**	1.32 (0.129)	1.09–1.60	0.005	1.34 (0.136)	1.10–1.63	0.004
**Previous mental health diagnosis**	1.19 (0.140)	0.95–1.50	0.133			
**Renter**	1.23 (0.169)	0.94–1.61	0.135			
**Smoker**						
Never	1.00					
Past	1.08 (0.116)	0.88–1.33	0.472			
Current	1.28 (0.362)	0.74–2.23	0.379			
**Education**						
No school qualification, school or intermediate, HSC or leaving	1.00					
Trade, apprentership, certificate or diploma	1.12 (0.193)	0.80–1.57	0.522			
University	0.88 (0.125)	0.66–1.16	0.357			
**Direct fire^b^**						
1.Any exposure	1.54 (0.146)	1.28–1.85	<0.001	1.40 (0.137)	1.15–1.69	0.001
2. Scale–None	1.00					
Mild	1.48 (0.144)	1.22–1.79	<0.001	1.34 (0.135)	1.10–1.63	0.004
Severe	1.98 (0.351)	1.40–2.80	<0.001	1.81 (0.326)	1.27–2.58	0.001
3. Cumulative	1.29 (0.068)	1.16–1.43	<0.001	1.22 (0.067)	1.10–1.36	<0.001
**Previous Fire^c^**						
1. Any exposure	1.22 (0.121)	1.01–1.49	0.039	1.36 (0.139)	1.11–1.66	0.003
2. Scale–None	1.00					
Mild	1.18 (0.120)	0.97–1.44	0.095	1.29 (0.135)	1.05–1.59	0.014
Severe	1.48 (0.245)	1.07–2.05	0.018	1.82 (0.312)	1.30–2.55	<0.001
3. Cumulative	1.16 (0.088)	0.999–1.35	0.051	1.24 (0.098)	1.06–1.45	0.006
Canberra bushfires 2003	1.07 (0.099)	0.89–1.28	0.452			

a Bivariate models included factors along with gender, age and gender^*^age interaction;

b *Direct exposure to fire in the current season was measured as: 1. Any exposure—yes/no if any direct exposures to fire were indicated; 2. Scale—a scale was created consisting of three levels of exposure—none (none or indirect), mild (classified as responses limited to being in an area with fire nearby, evacuation due to bushfire, area of significance lost other than home, family member was affected, home was affected while away), and severe (if experience included loss of or damage to property or direct contact with fire e.g., firefighter or protecting property); 3. Cumulative—the number of ways in which the participant has previously been exposed for fire were added.;*

c *Previous exposure to fire was measured as: 1. Any exposure–yes/no if any previous exposures were indicated; 2. Scale–a scale was created with three levels of exposure—None (not affected, effects were limited to health and/or smoke effects), mild (responses limited to voluntary evacuation, family or close friend affected, cancellation or alteration of holiday plans/events, business or work affected), and severe (if experience included forced evacuation, damage to or loss of property, firefighter, first responder, volunteer, protected property, alert to evacuate, worry about property or risk); 3. Cumulative–The number of ways in which the participant has previously been exposed for fire were added*.

### Medical Advice

Approximately 17% of people reported they sought advice from a health professional or medical facility in relation to their smoke-attributed symptoms (Table 7). The most commonly accessed source was a general practitioner (13.2%), followed by pharmacist (3.6%) and mental health professional (2%). Females were more likely to have sought health advice than males (21 vs. 11%, p <0.001).

**Table 7:** Proportion of surveyed ACT region residents that sought medical advice in relation to bushfire smoke attributed health conditions during the 2019–20 bushfire season.

	**Number seeking medical advice** ***n*** (%)		
	**Overall**	**Male**	**Female**
Medical advice from any source	332 (17.1)	80 (10.9)	251 (21.1)
General practitioner	275 (13.2)	65 (7.8)	209 (17.0)
Pharmacist	75 (3.6)	17 (2.1)	58 (4.7)
Mental health professional (e.g., psychologist)	42 (2.0)	6 (0.7)	36 (2.9)
Specialist	22 (1.1)	4 (0.5)	18 (1.5)
Other health professional	16 (0.8)	3 (0.4)	13 (1.1)
Emergency department	14 (0.7)	2 (0.2)	12 (1.0)
Hospital inpatient	8 (0.4)	3 (0.4)	5 (0.4)
24 h health advice hotline	6 (0.3)	3 (0.4)	3 (0.2)

*Females were more likely to have sought health advice than males (p < 0.001)*.

## Discussion

The extreme 2019–20 Australian bushfire season increased the burden on the health system (22). The current study indicates substantial self-reported effects of bushfire smoke on the ACT community's physical, mental health and sleep patterns, with almost all respondents reporting at least one negative health effect during this time. The most common symptoms (eye and throat irritation, cough) were consistent with other studies (23) and the known short-term effects of exposure to very high levels of PM_2.5_. Bushfire smoke-related air pollution reached hazardous levels in the ACT over a longer period and to higher levels than previously reported in other areas of south eastern Australia (23, 24). In the current ACT study, 97% of the sample reported at least one smoke-related health symptom, whereas prevalence of similar health conditions was 65.1% in the Hunter-New England area (25, 26), and 16.1% in a control population located in Hobart, Tasmania that was not affected by smoke.

The smoke-attributable health burden experienced in the ACT region is higher than other studies of the health effects of smoke (23). Other studies have relied on emergency presentations or hospital records to identify those experiencing negative health outcomes (8, 27–30), but the current study drew on self-reported effects from community members, only ~1% of whom presented at a hospital. Presentation measures only capture medical conditions severe enough for individuals to seek clinical care, therefore underestimating the total health burden. These measures may also miss mental health conditions, for which support may be sought elsewhere. The extent to which this study documents smoke effects is significant and concerning. The prevalence of health conditions for which formal medical advice was not sought highlights the importance of interventions to better prepare people on ways to reduce exposure during periods of hazardous air pollution and when to seek help. Barriers to accessing medical and psychological care were not investigated in this study, but future studies should examine this. Further epidemiological studies that accurately measure individual smoke exposure will be able to better characterize specific health impacts of severe smoke, or thresholds for these, and give more accurate advice about how and when individuals should protect their health.

Smoke exposure is known to exacerbate existing health conditions (14). Our finding that participants with an existing health condition had greater odds of experiencing more smoke-related health issues is consistent with this. Similarly, poorer self-rated health was associated with increases in both physical health and sleep-related symptoms. Sleep problems have been associated with both physical and mental health problems (31, 32) and physical and mental health are also strongly linked (33, 34). Future studies ought to examine the inter-relatedness of these health outcomes (14).

Several studies demonstrate the lasting psychological effects of bushfire exposure, including increased rates of depression, post-traumatic stress disorder (PTSD), and increased drug and alcohol use (35, 36). A year after fires in an Australian community, twice as many people (42%) were classified as “potential psychiatric cases” than in an unexposed comparison population (37). Most people do not develop psychological conditions following a natural disaster (38), but it is important to identify those at risk of doing so and to provide the support they require. Future research should also consider community factors such as community cohesion, competence and support (39) that may influence individual wellbeing, rather than focus solely on individual-level variables.

Women and men may experience, respond to, and recover from bushfires differently (40, 41). For example, women are more likely to report higher rates of PTSD (42, 43), and men to report an increase in alcohol abuse after experiencing bushfires (40, 44). In the current study, women reported higher rates of all physical health symptoms, poorer sleep, and mental health issues. Gender differences have been attributed to biological, social or situational factors (38, 40, 45).

Longer term mental health outcomes for communities exposed to bushfire are generally good, although a significant minority may experience persistent difficulties and vulnerability may increase with cumulative trauma exposures (44). In our study, previous exposure to fire was associated with greater odds of reporting poorer physical health outcomes and sleep disturbance, but not with mental health outcomes. More disrupted sleep for people who had previously been directly exposed to a bushfire event is possible, as the smoke may have triggered memories of that event. Direct exposure to fire is inherently difficult to understand as exposure severity and personal experience vary among individuals. To investigate this, we explored this measure in several ways–by assessing a binary (yes/no) response, a cumulative measure of the number of effects identified, or a ranking measure, in which some experiences were weighted more strongly than others. Unsurprisingly, people who were directly affected by fire had increased odds of physical, sleep, and negative mental health outcomes. For all of these outcomes, the pattern of relationship was similar, with higher odds ratios in the group ranked severe, compared to the mild group. The different ways of measuring exposure were all statistically significant, suggesting the link between direct experience of bushfire with adverse health outcomes is robust. In contrast, the cumulative measure showed consistently weaker odds ratios compared to other measures. This poses questions about the level of detail needed in measuring people's objective and subjective experience with bushfire, whether there are severity of impact thresholds for concern, and how a single measure might account for different experiences of a similar event (for example voluntary vs. forced evacuation). The complexity of this issue warrants more detailed study.

Although participants in this study were asked to focus on the effects of smoke (and not fire) specifically, it is likely some people were unable to separate the effects of smoke from other factors, such as heat stress or the direct effects of fire. Also, this study coincided with the early stages of the COVID-19 pandemic in Australia, and participants may have been unable to disentangle their feelings of distress and anxiety about the bushfires from the uncertainty of the developing pandemic.

During the 2019–20 bushfire season, there was community concern about whether smoke exposure would have long-term effects on health (46). This continues to be an area that is not well understood as only limited studies have explored long-lasting or delayed effects of smoke on health. Some have identified increased incidence of influenza (47) and impaired lung function (48) for months to years after a fire event. Canberra had experienced a severe bushfire in January 2003 and a survey 3 years after this disaster found that for over half the survey respondents (56.4%, n = 272) the bushfire did not have a lasting effect on their overall health and 2.5% (n = 12) reported that their overall health was better than before (18). However, 40.9% (n = 197) reported a lasting negative effect of the bushfire on their overall health. As such, we hypothesized that community members who had been exposed to the 2003 bushfires in Canberra may have been at greater risk of negative health effects during the 2019–20 fire season, particularly regarding negative mental health outcomes. However, this was not evident from our results, perhaps indicating that people were resilient and had had sufficient time to resolve any adverse reactions to the 2003 fires. Further longitudinal research is needed to understand the long-lasting, as well as the acute, health effects of bushfire and smoke exposure.

Rapid research during or immediately following natural disasters is challenging (49, 50). Here, a survey was designed to be deployed quickly to capture a broad snapshot of a population's health in response to a bushfire smoke event. Online data collection was suitable for a wide range of the population, but not when such as electricity or internet services are interrupted. The use of multiple sampling approaches captured experiences from a broad cross-section of the population, though the sample is not representative of the whole population as some groups such as the elderly, those with no fixed address, or those for whom English is not their primary language, may have been missed or underrepresented. Further, the focus here was on rapid and easy to measure self-report items with good face validity, rather than on more sophisticated measures of key constructs such as mental health. Despite these limitations, important trends concerning health and lifestyle burdens were identified, presenting an effective way to screen for specific areas or groups requiring more detailed examination. This method should, though, be refined to focus on the most pertinent and useful information to ensure ease of completion and associated rapid delivery of appropriate support and services to communities.

## Conclusions

Bushfire smoke can have considerable and underestimated effects on physical and mental health, beyond those associated with direct contact with fire and the acute effects of smoke inhalation. Greater understanding of mental health and long-term health effects is needed, particularly for at-risk groups, including parents, those with existing health conditions, or those who had previous exposure to fire and smoke. Improved public health communication is needed to strengthen individuals' ability to prevent harm and protect the health of themselves and their families for future events.

## Data Availability Statement

The raw data supporting the conclusions of this article will be made available by the authors, without undue reservation.

## Ethics Statement

The studies involving human participants were reviewed and approved by Human Research Ethics Committee of the Australian National University (protocol number: 2020/029). The patients/participants provided their written informed consent to participate in this study.

## Author Contributions

RR, AS, AC, BC, AL, JL, JR, ST, SV, and IW contributed to the conception and design of the study. ST and RR obtained ethics approval, conducted data collection, and database management. RR was responsible for data analysis and drafting of manuscript. All authors contributed to manuscript revision, read, and approved the submitted version.

## Funding

This research was funded by the Australian National University College of Health and Medicine. AC is supported by a National Health and Medical Research Council (NHMRC) Fellowship 1173146.

## Conflict of Interest

The authors declare that the research was conducted in the absence of any commercial or financial relationships that could be construed as a potential conflict of interest.

## Publisher's Note

All claims expressed in this article are solely those of the authors and do not necessarily represent those of their affiliated organizations, or those of the publisher, the editors and the reviewers. Any product that may be evaluated in this article, or claim that may be made by its manufacturer, is not guaranteed or endorsed by the publisher.
